# The contrasting role of climate variation on the population dynamics of a native and an invasive insect pest

**DOI:** 10.1371/journal.pone.0284600

**Published:** 2023-04-28

**Authors:** James Shope, Dean Polk, Carrie Mansue, Cesar Rodriguez-Saona

**Affiliations:** 1 Department of Environmental Sciences, New Jersey Climate Change Resource Center, Rutgers University, New Brunswick, New Jersey, United States of America; 2 Rutgers Specialty Crop Research and Extension Center, Rutgers University, Cream Ridge, New Jersey, United States of America; 3 Cooperative Extension of Atlantic County, Rutgers University, Mays Landing, New Jersey, United States of America; 4 P.E. Marucci Center, Rutgers University, Chatsworth, New Jersey, United States of America; University of Bari Aldo Moro, ITALY

## Abstract

Since 2008, spotted-wing drosophila, *Drosophila suzukii*, has become a major pest of soft, thin-skinned fruits in the USA, causing significant annual yield losses. Historically, the native blueberry maggot fly, *Rhagoletis mendax*, has been a key blueberry pest in eastern North America and a driver of insecticide usage. After its invasion in 2011 into New Jersey (USA), *D*. *suzukii* has supplanted *R*. *mendax* as the main target of insecticide applications in the state. However, the impact of *D*. *suzukii* on the native *R*. *mendax* has not been documented, particularly in relation to local climate. Historical monitoring data from New Jersey blueberry farms were used to assess the role of climate on *R*. *mendax* and *D*. *suzukii* populations. Seasonal trap captures of *R*. *mendax* adults have decreased after *D*. *suzukii* invasion, while *D*. *suzukii* trap captures have increased. Similarly, *D*. *suzukii* first captures have occurred earlier each year, while *R*. *mendax* has been captured later in the growing season. Winter freezing and summer growing degree days were found to significantly correlate with *D*. *suzukii* activity. Using downscaled climate simulations, we projected that *D*. *suzukii* will arrive in New Jersey blueberry fields up to 5 days earlier on average by 2030 and 2 weeks earlier by 2050 with warming temperatures, exacerbating yield losses and insecticide usage. As regional temperatures are projected to warm and the invasive range continues to expand, we predict the rate of phenological development of the invasive *D*. *suzukii* and its impact on native insects to change noticeably, bringing new challenges for pest management strategies.

## Introduction

For the last two centuries, worldwide insect invasions have increased [1], and developed countries are particularly vulnerable due to their considerable role in global trade [2]. These invasions have resulted in significant global economic costs, with estimated losses due to expenditures in pest prevention and control of more than US$ 1.288 trillion over the last 50 years [3]. One such invasive insect pest that has had a dramatic impact on fruit production worldwide is the spotted-wing drosophila, *Drosophila suzukii* (Matsumura) (Diptera: Drosophilidae) [4]. Native to southeast Asia, *D*. *suzukii* has become a major pest of soft, thin-skinned fruits (e.g., blueberries) in the USA and other parts of the world since 2008 because of its ability to lay eggs in ripening fruit [5–8], short generation times [4,9,10], high reproductive capacity and adaptation to variable environmental conditions [4,10,11], and the lack of effective natural enemies in the invaded regions [4,12]. Consequently, *D*. *suzukii* has caused significant annual yield losses to several crops such as blueberries, raspberries, strawberries, and cherries [13–16]. To manage this pest, farmers rely heavily on the repeated use of insecticides [4,17], although more sustainable alternative tactics such as cultural, behavioral, and biological control methods are currently under evaluation or, to some extent, have already been implemented [17,18].

Highbush blueberry (*Vaccinium corymbosum* L., Ericaceae) is a crop native to eastern North America (USA and Canada) that was first domesticated about 100 years ago in New Jersey (USA) [19]. New Jersey is currently one of the top six highbush blueberry-producing states in the USA, with a crop valued at US$ 78 million [20] and with Atlantic and Burlington counties accounting for more than 90% of the state’s blueberry production. Historically, the native blueberry maggot fly, *Rhagoletis mendax* Curran (Diptera: Tephritidae), has been a key pest of blueberries in its native range and the driver of insecticide applications during harvest [21,22]. However, since its invasion, *D*. *suzukii* has supplanted *R*. *mendax* as the main target of insecticide applications in most regions where these two pests co-occur [22], such as in New Jersey [23]. Nevertheless, the impact of the invasive *D*. *suzukii* on native blueberry pests, such as *R*. *mendax*, has yet to be documented, particularly in relation to its interaction with local climate conditions.

Both *D*. *suzukii* and *R*. *mendax* are direct pests of blueberries because females lay their eggs in the fruit and the larvae develop inside the fruit until pupation; however, differences in their biology and ecology have been observed. Specifically, *D*. *suzukii* can complete multiple, overlapping generations throughout the fruiting season [4] and overwinters as adults [4,14], whereas *R*. *mendax* is univoltine (produces one generation per year) [21] and overwinters as pupae in the soil [21]. Also, *D*. *suzukii* attacks blueberries earlier in the growing season, as soon as the fruit changes color from green to pink or pink to blue [5], while *R*. *mendax* females need to feed for 1–2 weeks after emergence for ovarian development prior to egg-laying (pre-ovipositional period) and lay their eggs in mature fruits [24]. Moreover, *D*. *suzukii* may either pupate inside of fruits or emerge to pupate in the soil [25], whereas *R*. *mendax* pupates exclusively in the soil [26]. Since only one *R*. *mendax* larva can survive per fruit, females oviposit a single egg inside a blueberry fruit and subsequently mark the fruit with a pheromone to avoid intraspecific competition [27,28]. In contrast, *D*. *suzukii* can lay multiple eggs per fruit and many offspring can complete development from a single fruit [29].

Because of these differences in biology and ecology between the native *R*. *mendax* and the invasive *D*. *suzukii*, we expect these pest species to respond differently to observed and projected future climate conditions. In this study, we used historical monitoring trap data collected from multiple blueberry farms in New Jersey to test the role of climate on the population dynamics of *R*. *mendax* and *D*. *suzukii*. In New Jersey, *D*. *suzukii* was first reported in 2011 [23]; thus, we focused on climatic variation before and after invasion. We first investigated historic (2004–2022) changes in mean seasonal (i.e., summer) trap captures (as a proxy of population size) and in the date of first trap capture (as a proxy of population activity) of both pest species. To analyze these changes, we then correlated the population-level parameters to climate conditions, such as winter temperatures, and insecticide use. Finally, we used downscaled climate model simulations and population-level data to project potential future annual first capture dates of *D*. *suzukii* under two climate change scenarios. As regional temperatures are projected to warm [30,31], we predict the rate of phenological development of *D*. *suzukii* to change noticeably, bringing new challenges for pest management strategies.

## Materials and methods

### Trap design and monitoring

Historic trapping data (2005–2022) for *R*. *mendax* and *D*. *suzukii* and insecticide spray records (2010–2022) were obtained from the Fruit Integrated Pest Management (IPM) program at Rutgers University (New Jersey, USA) (for information on the program please see the Rutgers Fruit IPM website [32]). Over this period, the Rutgers Fruit IPM program provided monitoring services for various insect pests, including *R*. *mendax* and *D*. *suzukii*, to an average of 41 highbush blueberry farmers in Burlington and Atlantic counties (New Jersey, USA). The timing of trap deployment for adults of both *R*. *mendax* and *D*. *suzukii* are based on the annual calendar, with traps being placed around the US Memorial Day which normally coincides with the time when the ‘Duke’ blueberry variety, an early variety, starts to turn blue. Therefore, this program has a unique statewide data set that captures changes in the population dynamics of *R*. *mendax* and *D*. *suzukii* before and after the invasion of *D*. *suzukii*.

The number of *R*. *mendax* monitoring sites (blueberry fields) increased from an average of 37 in mid- to late 2000s (~1 per farm) to 72 in mid- to late 2010s (1.5–2 per farm). Monitoring of adult flies utilized sticky traps baited with ammonium acetate (Pherocon^®^ AM; Trécé, Adair, OK) [33,34]. Traps (1 or 2 per site) were hung in a “V” orientation within the top of the blueberry canopy and at the edge of the field, near a wooded border [35,36]. Each year, the traps were deployed at least 2 weeks before the expected first emergence of the flies and were replaced every 2 weeks. The traps were checked weekly for *R*. *mendax* adults from the time of deployment until end of harvest (i.e., ~3 months, from early-June to end of August).

For *D*. *suzukii*, the number of monitoring sites (blueberry fields) was on average 54 (~1 per farm). The trapping method for *D*. *suzukii* adults was modified to reflect advancements in monitoring tools. From 2012 to 2014, adults were monitored using clear, plastic 0.95-L deli cup traps baited with 150 mL of apple cider vinegar with 0.1% soap (unscented Seventh Generation soap) to break the surface tension [23]. The trap had two (2.5-cm diameter) round holes on opposite sides, and the holes were 7 cm from the bottom of the trap and were covered with a ~3-mm mesh for fly entry. Each week, the traps were replaced with new apple cider vinegar bait. From 2015 to 2019, the same deli cup traps were used but baited with the fermentation volatile-based commercial Scentry^®^ Spotted Wing Drosophila lure (Scentry Biologicals, Inc., Billings, MT) and contained 150 mL of soapy water as a drowning solution. The Scentry^®^ lure is more effective at capturing *D*. *suzukii*, i.e., attracts more flies and provides earlier detection than the apple cider vinegar bait [37,38]. From 2019 to 2022, the deli cup traps were replaced by red sticky traps that were baited with the Scentry^®^ lure [39]. The red sticky traps are equally as effective in capturing *D*. *suzukii* but are easier to process than the deli cup traps [40]. The Scentry® lures were replaced every 4 weeks. Both deli cup and red sticky traps were hung from a branch at the bottom of the blueberry bush about 0.5–1 m from the ground, along the edges of the field facing wooded borders. One trap was placed per site, and traps were checked weekly for *D*. *suzukii* adults from early June until end of August.

### Trapping dataset information

#### Trapping data

Two datasets were utilized to discern changes in *R*. *mendax* and *D*. *suzukii* population-level parameters. The first dataset was the annual first trap captures of *R*. *mendax* and *D*. *suzukii* as a proxy for population activity in each year. These data indicate the first Julian day each year when these two species were detected in traps in blueberry fields throughout New Jersey (USA). For the native *R*. *mendax*, we used records of first catch from 2005 to the present day (2022 at the time of writing), only missing the year 2014, to capture the period before and after *D*. *suzukii* invasion. For the invasive *D*. *suzukii*, we used the first capture data from 2012 to present day.

The second dataset was the weekly average number of seasonal (i.e., summer) captures per trap as a proxy for population size in a given year. For both insects, the weekly average was calculated as the average capture across all traps across all blueberry fields for the given week. The trapping season for *R*. *mendax* extended from 01 June to 27 August and from 26 May to 28 August for *D*. *suzukii*. For *R*. *mendax*, the peak weekly average capture number was extracted from the trap captures dataset, representing the week range when the average number of captured individuals was the highest for the season. Because *D*. *suzukii* has multiple generations per year, population numbers typically increase throughout the growing season, peaking after trapping has completed each year. Therefore, for *D*. *suzukii*, we used the mid-trapping season average number of captures, averaging the weekly average captures for the month of July across the state of New Jersey each year, to represent population size. In the absence of a peak capture metric, it was determined that an average over the course of a month would account for any variabilities tied to a specific date while representing high activity in blueberry fields during the middle of the season. To facilitate a comparison of the population metrics between the two pest species, we normalized (z-score transformation and mean centering) each population dataset prior to analysis.

Each trapping data metric, average trap captures and the date of first capture, was evaluated using an outlier analysis to identify erroneous data points due to potential mistakes during field data handling and recording. The time series of first catch days was extracted from Rutgers University’s *The Blueberry Bulletin* [41], which provides weekly information about insect activity in blueberry farms across the state of New Jersey. Gaps in this time series were filled using supplemental information from the Rutgers Fruit IPM program [32]. In most cases, the fill dates were consistent with the range provided by *The Blueberry Bulletin*. However, a few were identified by entomological and pest management expert assessment as too late in the season to be credible and likely recorded in error. We used an outlier analysis to statistically identify these points via a quartile analysis whereby the datapoints more than 1.5 interquartile ranges above the upper quartile or below the lower quartile were identified and removed. In practice, we identified four outliers that were removed from the analysis which included the first capture date of *R*. *mendax* in 2014, the trap capture numbers of *R*. *mendax* in 2009 and 2013, and the trap capture number of *D*. *suzukii* in 2019. The full data sets, prior outlier analysis, have been provided (please see Data Availability).

#### Trapping data analyses

We compared the first capture dates of *R*. *mendax* and *D*. *suzukii* by computing Pearson’s correlation coefficient (*r*) between the *R*. *mendax* first capture dates and *D*. *suzukii* first capture dates of the prior year. We predicted that the effect of *D*. *suzukii* activity on *R*. *mendax* first capture date would be noticeable the following year, i.e., after the yearly competition for resources, which has been observed in other interspecies interactions [42]. For example, the *R*. *mendax* first capture data for 2022 was compared against *D*. *suzukii* first capture in 2021. We also limited the correlation to the years 2016 to 2022 for *R*. *mendax* and 2015 to 2021 for *D*. *suzukii* because we assumed that the first two years (2012 and 2013) of the *D*. *suzukii* first captures are establishment years (following Leach et al. [38]), and thus, they do not represent the *D*. *suzukii* capture trend and its relation to *R*. *mendax* first captures in subsequent years. Also, 2015 represented a shift to using the Scentry^®^ lure in traps, so limiting the analysis to after its implementation removes potentially confounding effects from changing bait types. Correlations for *R*. *mendax* peak capture numbers and *D*. *suzukii* midseason capture numbers were also calculated for the years after 2014 to account for the change in *D*. *suzukii* trap bait.

### Insecticide information

#### Insecticide data

To assess whether trends in *R*. *mendax* and *D*. *suzukii* population size are related to the number and frequency of insecticide applications, we compiled the annual number of post-bloom insecticide sprays [from fruit set (June 1) until end of harvest (August 31)] for 8 blueberry farms in New Jersey that are part of the Fruit IPM program. Note that *R*. *mendax* and *D*. *suzukii* are the main pest targets of post-bloom insecticide applications. The spray record extended from 2010 to 2021 to account for the period after *D*. *suzukii* invasion, with most farms missing data for the year 2011. The spray numbers were normalized by farm to account for differing acreage and number of individual fields.

#### Insecticide data analyses

The normalized spray numbers were compiled into an average annual time series to represent the region and compared with *R*. *mendax* and *D*. *suzukii* midseason capture numbers. Correlations were only computed for *D*. *suzukii* after establishment (2015 to 2021) and for *R*. *mendax* from 2012 to 2021, removing 2010 and 2013 as abnormally high outliers in the *R*. *mendax* capture number data. A new outlier analysis using the interquartile range approach was conducted for the shortened time series that were compared with spray values. While the *D*. *suzukii* outliers remained the same (just 2019), 2010 was identified as an additional outlier in the shortened *R*. *mendax* time series and removed in this analysis. In the correlation analysis described in the results, 2010 overtly dominated the correlation, and its removal similarly removed any trend. Additionally, 2011 was removed from this analysis due to gaps in spray data.

### Climate data information

#### Climate data

To determine whether climate conditions explain changes in *R*. *mendax* and *D*. *suzukii* population activity, daily measurements of maximum, minimum, and average temperature; mean rainfall; dewpoint temperature; and vapor pressure deficit (Table 1) were retrieved from Oregon State University’s Parameter-elevation Regressions on Independent Slopes Model (PRISM [43], data created 3 June 2022, accessed 7 June 2022) for January 2004 to May 2022. The model output location was selected to be coincident with the Hammonton (Atlantic Co.), New Jersey, weather station (NWS Coop 283662) by using the interpolation option through the PRISM interface that uses an inverse-distance squared weighting of surrounding model grid cells to provide more site-relevant climate variable output. This location was selected because most of the blueberry production in New Jersey is concentrated in Hammonton. We decided that the PRISM data were appropriate for this study, as PRISM provides continuous, long-term information about the climate and daily weather variables within the area. There are two meteorological observational platforms in the region around Hammonton, New Jersey; however, the data record of one station operated by the Office of the New Jersey State Climatologist is shorter than the *R*. *mendax* capture records, and the other (NWS Coop 283662) appears to have been taken offline at the end of 2021. Therefore, to ensure that consistent and easily accessible data products were used in the analysis and for application of *D*. *suzukii* monitoring moving forward, it was decided that PRISM offered the best integration of observation calibration and record continuity.

**Table 1:** Summary of seasonal climate variables extracted from station data for comparison with *Rhagoletis mendax* and *Drosophila suzukii* annual first capture dates.

Variable	Description
*D*_*Freeze*_	Seasonal number days with average temperatures below freezing (0°C [32°F])
*D*_*50*_	Seasonal number days with average temperatures above 10°C (50°F), when flight is possible for *D*. *suzukii* [63]
*T*_*avg*_ (°F)	Seasonal average air temperature
*D*_*precip*_	Seasonal number days with average precipitation above 0.25 inches
*P*_*avg*_ (inches)	Seasonal average precipitation
*DD*_50_	Seasonal degree days, base 10°C (50°F)
*DD*_32_	Seasonal degree days below freezing, base 32°F (0°C)
*T*_*dp*_ (°F)	Seasonal average dewpoint temperature
*VPD*_*min*_	Seasonal average of minimum daily vapor pressure deficit
*VPD*_*max*_	Seasonal average of maximum daily vapor pressure deficit

The daily weather measurements were compiled into multiple seasonal statistics for winter (December–February), spring (March–May), summer (June–August), and fall (September–November). Summer and fall values were calculated for the prior year relative to the capture date (e.g., the capture date data in 2021 were compared to summer and fall conditions from 2020). Winter values were calculated for the preceding December–February conditions (e.g., the 2021 capture data were compared against the December 2020–February 2021 winter data). Spring values were computed for the coincident year (e.g., the 2021 capture data were paired with the 2021 spring condition data). The assumption was that the preceding summer through winter conditions may influence the first capture date in the spring/early summer, but the “year-of” spring conditions are more influential than small influences from the prior spring. The computed seasonal climate statistics are listed in Table 1 and were computed for each season on an annual basis.

### Climate data analyses

For blueberry growers, the first capture date each year is an important metric by which to start insecticide spray cycles to manage *R*. *mendax* and *D*. *suzukii*. Based on the biology of these insects, it is likely that year-to-year variations in first capture dates are influenced by environmental factors, such as winter/spring temperatures [44]. Following a method similar to that in Leach et al. [44], we computed correlations of *R*. *mendax* and *D*. *suzukii* seasonal trap captures and first catch data from 2005 to 2022 and 2015 to 2022, respectively, with seasonal meteorological variables listed in Table 1 to help elucidate which conditions in New Jersey may affect these two pests. To capture the effects of the *D*. *suzukii* invasion on *R*. *mendax* population dynamics, three separate correlation analyses were conducted, as follows: prior to *D*. *suzukii* establishment (2005–2013), after *D*. *suzukii* establishment (2015–2022), and the entire time series (2005–2022). Finally, for both *R*. *mendax* and *D*. *suzukii*, the prior year’s capture number was also investigated to determine if relative changes in population size influenced the first catch of the subsequent year.

### Predictive linear model development

We developed simple linear models to project first *D*. *suzukii* capture each year (Table 2) based on winter and prior summer temperature conditions. The first goal of this effort was to produce an annual predictive model that can be used to help project annual *D*. *suzukii* arrival in New Jersey blueberry fields for IPM usage. The second was to use the degree day model to inform how future climate change may affect *D*. *suzkii* to arrival dates over the next 20–30 years. No predictive model was developed for *R*. *mendax* as only prior summer *P*_*avg*_ was found to be a potential predictor after *D*. *suzukii* establishment. While *P*_*avg*_ can be calculated from projected climate data, there was not a clear biological cause that would indicate that this relation was not spurious. Also, after *D*. *suzukii* invasion, *R*. *mendax* activity was likely driven by other environmental stressors (such as competition with *D*. *suzukii*). Therefore, we elected not to develop a predictive model for *R*. *mendax* first captures.

**Table 2:** Linear model fit parameters of *Drosophila suzukii* first capture dates.

Linear Model	Coefficient(s)	Intercept	*r*^*2*^ (Adjusted *r*^*2*^)	RMSE (days)	AIC
Winter *DD*_32_	0.079	136.636	0.72 (0.69)	6.034	53.2
Summer *DD*_50_	−0.083	342.626	0.74 (0.69)	5.792	52.5
Winter *DD*_32_ + Summer *DD*_50_	0.0378_Winter *DD32*_, −0.0490_Summer *DD50*_	257.043	0.78 (0.69)	5.849	53.2

Based on our analysis (see Results), winter *DD*_32_, summer *DD*_50_, summer *T*_*avg*_, and summer *T*_dp_ (see variable definitions in Table 1) had significantly high correlations with *D*. *suzukii* first capture dates. Therefore, these variables were used as the basis of developing predictive first capture models of *D*. *suzukii*. Summer *T*_*avg*_ had the same correlation with *D*. *suzukii* capture dates as summer *DD*_50_, so *T*_*avg*_ was excluded from this effort. Similarly, summer *T*_*dp*_ was excluded as the utilized downscaled future climate projections do not provide metrics for *T*_*dp*_ or atmospheric moisture (humidity). Multivariable models were also tested but most performed similar to or worse than the single-variable models.

One multivariable model that did improve fit combined winter *DD*_32_ and summer *DD*_50_ (Table 2), which resulted in a larger confidence interval. This multiple linear model is included in the Results and Discussion. The summer *DD*_50_ model produced a similar fit, the smallest root mean squared error, and the lowest (best) Akaike Information Criterion (AIC), explaining the most variation with the fewest independent variables, when compared to the other models. Additionally, we elected to calibrate the model using *D*. *suzukii* first catch data from 2015 to 2022 to remove establishment years, outlier years, and years where a different lure was utilized.

Future climate temperature data were input into these models to project how *D*. *suzukii* capture dates may change with a warming climate. Future daily meteorological data were accessed through the NOAA Regional Climate Center’s Applied Climate Information System [45], which has downscaled regional climate projections for the northeast USA for the following two emission scenarios: Representative Concentration Pathways (RCPs) 4.5 and 8.5 [46,47]. RCP 4.5 and RCP 8.5 represent an intermediate and high greenhouse gas emission scenario, respectively, modeled from 1950 through 2100. Seasonal temperature statistics were computed from daily average temperature output of 31 Coupled Model Intercomparison Project 5 general circulation models downscaled to the New Jersey Region following the Localized Constructed Analogues methodology [48]. These outputs were combined into seasonal weighted means on an annual basis.

## Results

### Trap captures over time

**Fig 1:**
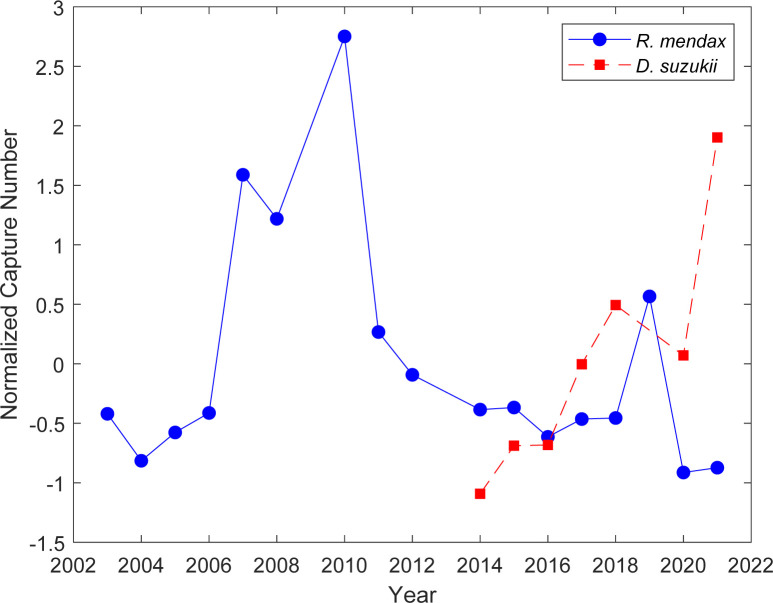
The annual normalized mean peak capture number of *Rhagoletis mendax* (solid blue line with blue circles), and mean midseason capture number of *Drosophila suzukii* (dashed red line with white squares), in highbush blueberry farms in New Jersey (USA).

*Rhagoletis mendax* peak adult captures varied substantially from 2004 to 2021 (0.03 to 2.11 average weekly capture across all traps), with maximum captures in 2010 (Fig 1). Since 2012, the average number of captures followed a decreasing trend with an exception in 2019 (Fig 1). Since its introduction, *D*. *suzukii* midseason catch numbers followed a near-linear increase from 2014 to 2021, peaking in 2021 at an average midseason capture of 17.21 *D*. *suzukii* adults (after removing the outlying 2019 value). A correlation between these trends was not found to be significant due to the variations in the data.

**Fig 2:**
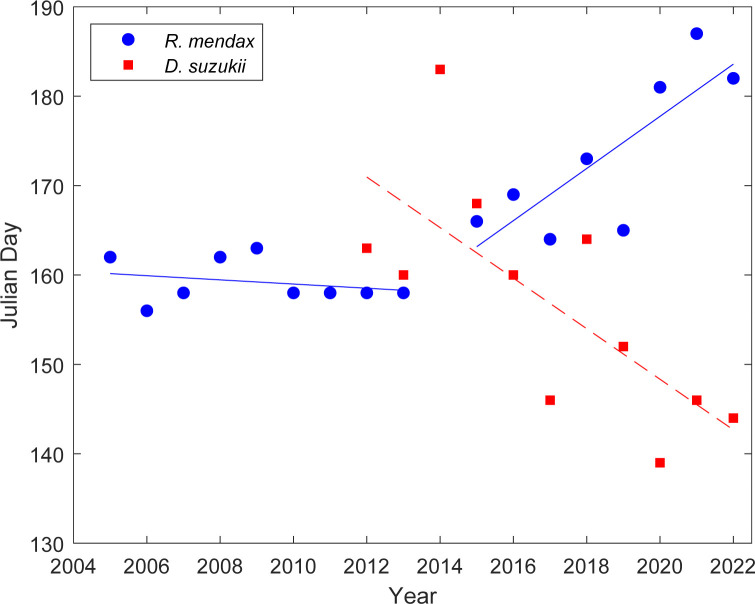
Annual first capture dates in Julian days of *Rhagoletis mendax* and *Drosophila suzukii*. Blue circles represent observed *R*. *mendax* capture date, red squares represent observed *D*. *suzukii* capture date, the solid blue line is a linear fit of the *R*. *mendax* first capture dates for the period of 2005 to 2013 and 2015 to 2022, and the dashed red line is a linear fit of the *D*. *suzukii* from 2012 to 2022. The correlation coefficient (*r*) between annual *R*. *mendax* captures and annual *Drosophila suzukii* captures of the prior year is *r* = −0.84 with a *P* = 0.019 indicating significant correlation.

Prior to 2013, the *R*. *mendax* first capture date was consistent within the range of 156–163 Julian days (Fig 2). Starting in 2015, however, *R*. *mendax* was noticeably caught later and later in the year, reaching as late as 187 days in 2021 (Fig 2). Conversely, while there was some variability, the first capture dates of *D*. *suzukii* have generally occurred earlier each year in a near-linear fashion from 2015 to 2022 (i.e., after population establishment) at 168 to 144 Julian days respectively. Pearson’s correlation showed that the first capture dates of *R*. *mendax* and *D*. *suzukii* of the prior year have a significant inverse relationship (*r* = −0.84, *P* = 0.019) for the years of 2015 to 2022, suggesting that after its establishment, *D*. *suzukii* activity might be affecting *R*. *mendax* first captures.

### Effects of insecticides

**Fig 3:**
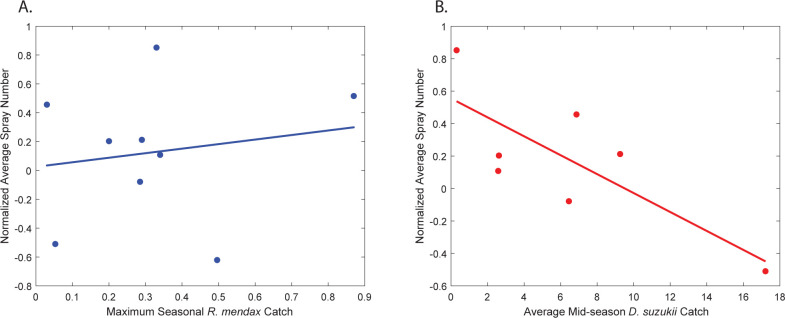
Normalized average annual post-bloom insecticide sprays compared to A. annual peak trap captures of *Rhagoletis mendax* (blue, with linear trend) and B. average midseason trap captures of *Drosophila suzukii* (red, with linear trend). *Drosophila suzukii* captures are significantly correlated with spray numbers at *r* = −0.78 with *P* = 0.04.

To rule out whether insecticide applications were responsible for the observed changes in *R*. *mendax* and *D*. *suzukii* population sizes, we determined Pearson’s correlations between *R*. *mendax* and *D*. *suzukii* trap captures and the number of post-bloom insecticide sprays. *R*. *mendax* peak captures did not correlate with the normalized average post-bloom spray numbers (*r* = 0.17, *P* = 0.67) (Fig 3A), indicating that increases in insecticide sprays in recent years due to *D*. *suzukii* invasion do not seem to affect *R*. *mendax* populations despite the decline in trap captures. In contrast, *D*. *suzukii* midseason capture numbers were significantly correlated with the normalized average number of post-bloom insecticide sprays each year (Fig 3B). Years with a greater number of sprays have lower average midseason *D*. *suzukii* trap captures than years with fewer sprays (*r* = −0.78, *P* = 0.04).

### Effects of climate

The mean trap captures (population size) and date of first trap capture (population activity) for *R*. *mendax* and *D*. *suzukii* were correlated with seasonal climate variables listed in Tables 3 and 4.

**Table 3:** Selected Pearson correlation coefficients (*r*) of *Rhagoletis mendax* annual maximum capture number and *Drosophila suzukii* average midseason capture number with seasonal climate variables. Three time periods are presented for *R*. *mendax* to correspond to before and after *D*. *suzukii* invasion and across the entire time period.

Variable	*D*. *suzukii*	*R*. *mendax* 2005–2021	*R*. *mendax* 2005–2013	*R*. *mendax* 2015–2021
Winter *T*_*avg*_	−0.04	−0.28	−0.23	−0.30
Winter *D*_*Freeze*_	−0.14	0.33	0.26	0.33
Winter *DD*_32_	−0.40	−0.11	−0.05	−0.28
Winter *D*_50_	−0.39	−0.16	−0.02	−0.16
Winter *D*_*precip*_	0.39	0.20	0.53	−0.05
Spring *T*_*avg*_	0.36	0.37	0.30	0.39
Spring *D*_50_	0.50	0.20	0.27	0.11
Spring *D*_*precip*_	−0.36	0.11	0.63	0.07
Summer *T*_*avg*_	0.56	−0.20	−0.33	−0.07
Summer *T*_*dp*_	0.75	−0.06	−0.29	−0.03
Summer *DD*_50_	0.56	−0.20	−0.33	−0.07
Summer *P*_*avg*_	0.42	−0.10	0.05	−0.53
Summer *D*_*precip*_	0.20	0.13	0.33	−0.71
**Prior Year Catch**	0.85	0.33	0.08	−0.21

*No correlations were found to be significant with *P* ≤ 0.05.

**Table 4:** Selected Pearson correlation coefficients (*r*) of *Rhagoletis mendax* and *Drosophila suzukii* annual first capture dates with seasonal climate variables. Three time periods are presented for *R*. *mendax* to correspond to before and after *D*. *suzukii* invasion and across the entire time period.

Variable	*D*. *suzukii*	*R*. *mendax* 2005–2022	*R*. *mendax* 2005–2013	*R*. *mendax* 2015–2022
Winter *T*_*avg*_	−0.52	0.14	−0.18	0.08
Winter *D*_*Freeze*_	0.71	−0.15	0.11	−0.13
Winter *DD*_32_	**0.85**	0.14	−0.31	0.36
Winter *D*_50_	0.21	0.14	0.05	−0.14
Winter *D*_*precip*_	0.28	0.14	−0.15	0.02
Spring *T*_*avg*_	−0.38	−0.13	−0.48	0.10
Spring *D*_50_	−0.39	−0.01	−0.52	0.03
Spring *D*_*precip*_	0.60	0.11	0.64	−0.48
Summer *T*_*avg*_	−**0.86**	0.04	−0.52	0.36
Summer *T*_*dp*_	−**0.87**	0.29	−**0.76**	0.62
Summer *DD*_50_	−**0.86**	0.04	−0.52	0.36
Summer *P*_*avg*_	−0.04	0.35	−0.28	**0.73**
Summer *D*_*precip*_	−0.35	0.25	−0.10	0.64
**Prior Year Catch**	−0.59	−0.33	0.22	−0.05

*Bolded numbers indicate significant correlations with *P* ≤ 0.05.

### Rhagoletis mendax

Prior to *D*. *suzukii* establishment (2005–2013), *R*. *mendax* mean trap captures were insensitive to all explored seasonal climate variables, but the strongest correlation was found to be with spring *D*_*precip*_, with a higher *D*_*precip*_ correlating with higher trap captures that year (Table 3). Additionally, summer *T*_*dp*_ had also a significant correlation with *R*. *mendax* first capture dates (Table 4), showing the consistency of this effect across the two population-level metrics. After *D*. *suzukii* establishment (2015–2022), again, no climate parameter was significantly correlated with seasonal *R*. *mendax* capture numbers. Summer *D*_*precip*_ generated the strongest insignificant correlation. (Table 3), with more rainy days the one year correlated with reduced *R*. *mendax* population size in the following year. Moreover, *R*. *mendax* first capture date was significantly correlated with summer *P*_avg_ (Table 4), with higher rainfall correlated with delayed first *R*. *mendax* capture. Across the entire *R*. *mendax* time series (2005–2021), trap capture numbers again did not significantly correlate with any climate variable or prior year capture (Table 3), and no climate factor correlated significantly with first capture date either (Table 4).

### Drosophila suzukii

**Fig 4:**
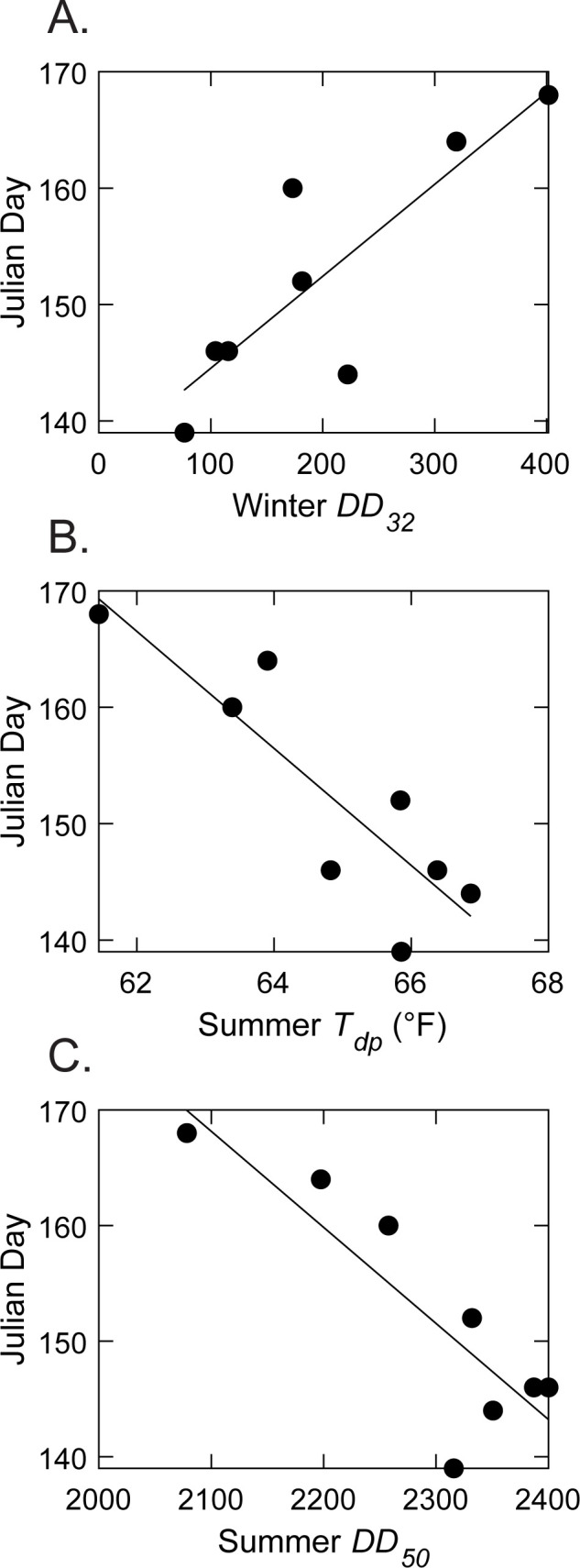
Comparison trend of *Drosophila suzukii* first capture days to A. winter *DD*_32_, B. prior summer *T*_*dp*_, and C. prior summer *DD*_50_ from 2015 to 2022.

*Drosophila suzukii* midseason capture numbers did not significantly correlate with any explored climate variable. The strongest insignificant correlation for midseason captures was summer *T*_*dp*_ (*r* = 0.75, Table 3), linking greater prior summer atmospheric moisture (proxied by *T*_dp_) to greater *D*. *suzukii* population size in the following year. First *D*. *suzukii* capture dates were significantly correlated with winter *DD*_32_, summer *T*_*avg*_, summer *DD*_50_, and summer *T*_*dp*_ (Table 4). Broadly, if the amount of time below freezing temperatures was higher than normal during a particular winter, the first catch tended to be later in the following year (Fig 4). Conversely, higher summer temperatures and greater summer atmospheric moisture significantly correlated with earlier first catch dates the following year (Fig 4). Although not significant, midseason trap captures of the prior year weakly and insignificantly correlated with the following year’s first catch date and midseason capture numbers (Tables 3 and 4), indicating that a larger population the prior year could possibly lead to earlier catch dates in the following year. Regardless, it appears that the *D*. *suzukii* first catch date was more related to environmental variables rather than to the prior year’s population size.

**Fig 5:**
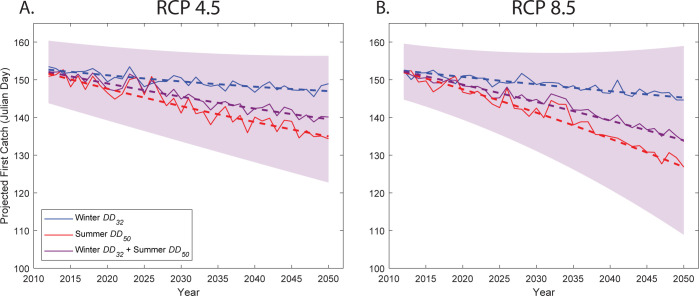
Projected *Drosophila suzukii* capture date based on three linear models corresponding to winter *DD*_32_ (blue), prior summer *DD*_50_ (red), and a multiple linear model incorporating both winter *DD*_32_ and prior summer *DD*_50_ (purple) for the following two climate change scenarios: A. RCP 4.5 and B. RCP 8.5. Dashed lines represent a quadratic fit of each model output. The purple shaded region represents a quadratic fit to the range of the 95^th^ percent confidence bounds of the combined winter *DD*_32_ and prior summer *DD*_50_ linear model. All data shown are model outputs driven by climate model output and may not match observed capture dates.

*Predictive model*. With climate change over the next century, the first catch day for *D*. *suzukii* is projected to occur earlier in the year (Table 5). Using a 2020 capture date of 139 Julian days (18 May) as a baseline and the intermediate greenhouse gas emission scenario RCP 4.5 [46], a linear model based on winter *DD*_32_ showed that the mean first *D*. *suzukii* capture will be approximately 1.8 ± 5.6 days earlier by 2030 and about 4.1 ± 6.2 days earlier by 2050 than the modeled mean for the period of 2011–2030 (Table 5, Fig 5A). Under the high greenhouse gas emission scenario RCP 8.5 [47], *D*. *suzukii* first catch is expected to be about 1.6 ± 5.7 days earlier by 2030 and about 5.2 ± 6.9 days by 2050 than the modeled mean for the period of 2011–2030 (Table 5, Fig 5B).

**Table 5:** Linear model projections and changes of *Drosophila suzukii* annual first capture days relative to the observed 2020 first capture date under RCP 4.5 and RCP 8.5 for the years 2030 and 2050 with 95^th^ percentile intervals.

Projection	Linear Model	Projection Year	Projected First Capture Julian Day ± 95th Percentile Confidence Interval	Change from 2020 (Days)
RCP 4.5	Winter *DD*_32_	2030	137.2 ± 5.6	−1.8
		2050	134.9 ± 6.2	−4.1
	Summer *DD*_50_	2030	134.3 ± 7.5	−4.7
		2050	125.9 ± 11.5	−13.1
	Winter *DD*_32_ + Summer *DD*_50_	2030	135.4 ± 10.4	−3.6
		2050	129.3 ± 17.7	−9.7
RCP 8.5	Winter *DD*_32_	2030	137.4 ± 5.7	−1.6
		2050	133.8 ± 6.9	−5.2
	Summer *DD*_50_	2030	133.7 ± 8.2	−5.3
		2050	118.6 ± 16.2	−20.4
	Winter *DD*_32_ + Summer *DD*_50_	2030	135.1 ± 11.7	−3.9
		2050	124.5 ± 26.3	−14.5

Under the RCP 4.5 scenario, the linear model based on summer *DD*_50_ showed that first catch would be approximately 4.7 ± 7.5 days earlier by 2030 and 13.1 ± 11.5 days earlier by 2050 compared to the modeled 2011–2030 mean (Table 5, Fig 5A). In the RCP 8.5 scenario, the median first capture date would be 5.3 ± 8.2 days earlier by 2030 and 20.4 ± 16.2 days earlier by 2050 (Table 5, Fig 5B).

The combined multiple linear model incorporating both winter *DD*_32_ and summer *DD*_50_ projects that *D*. *suzukii* first catch would be approximately 3.6 ± 10.4 days earlier by 2030 and 9.7 ± 17.7 days earlier by 2050 under RCP 4.5 (Table 5, Fig 5A). Under RCP 8.5, the first capture dates are 3.9 ± 11.7 days earlier by 2030 and 14.5 ± 26.3 days earlier by 2050 (Table 5, Fig 5B). Finally, Table 5 includes projected first capture dates relative to the observed 2020 first capture date of 139 Julian days.

## Discussion

Using historic statewide trapping data collected from commercial highbush blueberry farms in New Jersey (USA), we uncovered two clear, contrasting trends in the seasonal population size and activity of two major frugivorous pests of blueberries, namely, the native *R*. *mendax* and the invasive *D*. *suzukii*. Since the invasion of *D*. *suzukii* in 2011, 1) the seasonal (i.e., summer) catch numbers of *R*. *mendax* adults are declining, while the seasonal catch numbers of *D*. *suzukii* are increasing; and 2) the first capture dates of *R*. *mendax* have been occurring later in the season, while the first capture dates of *D*. *suzukii* have been occurring earlier in the season.

Since its invasion, *D*. *suzukii* seasonal populations have continued to increase in highbush blueberry farms in New Jersey; a similar pattern was reported in lowbush blueberries in Maine, another northeastern state in the USA [49]. In New Jersey, none of the climate variables that we tested correlated with *D*. *suzukii* population size. However, as expected, insecticides negatively correlated with *D*. *suzukii* population size, with more sprays during harvest in blueberry fields resulting in lower trap captures. In a previous study, Rodriguez-Saona et al. [50] also showed that repeated insecticide applications in blueberry fields reduce *D*. *suzukii* trap captures. Because insecticide applications in New Jersey blueberry farms increased after *D*. *suzukii* invasion, neither climate variables nor insecticide applications explain the observed increase in *D*. *suzukii* populations. In contrast, it is likely that biotic factors such as *D*. *suzukii* interactions with other species, or their lack thereof, might explain this pattern. As stated by the “enemy release” hypothesis [51], when *D*. *suzukii* arrived in the USA, it left behind its natural enemies (i.e., larval parasitoids) in its native country, Asia, that are adapted to *D*. *suzukii* biology and thus are more effective than those parasitoids in the introduced range [52,53]. The low success rate of parasitoids in the invaded territories is explained by a stronger immune response of *D*. *suzukii* larvae to parasitism than that of native drosophilids [54–57]. This lack of effective biological control agents may explain, at least partially, the increase in *D*. *suzukii* population size seen in our study since its invasion.

As *D*. *suzukii* population size increased through the years, adults have been caught in traps earlier. That earlier activity results in more generations per season and therefore larger populations. Although winter conditions induce the appearance of *D*. *suzukii* adult winter morphs that are darker and can tolerate brief exposures to low temperatures [58–60], harsh winters still act as a bottleneck in temperate regions by lowering its population size [61]. In fact, trapping data for the northeast USA show very few captures of *D*. *suzukii* adults during the winter compared with those during summer months [62], which is a pattern that could be due to lower population sizes but also to lower adult activity and a lack of response to volatile cues from traps by the winter compared with that of the summer morphs [63]. Nevertheless, we expect that greater numbers of overwintering *D*. *suzukii* adults could result in larger populations in the spring because of a greater likelihood of overwinter survival, leading to earlier trap captures, in contrast with Briem et al. [64]. Despite the findings of Leach et al. [61], there was not a significant correlation between current year and prior *year D*. *suzukii* capture numbers, though this was likely due to few data points after accounting for outliers in the *D*. *suzukii* capture data. Unlike our results for seasonal (i.e., summer) population size, we found that *D*. *suzukii* first capture date is affected by climate variables (winter freezing temperatures delayed first captures), while prior summer temperatures advanced first captures. Past studies have linked winter freezing days and warm (>10°C [50°F]) winter and spring days to *D*. *suzukii* first captures [44]. In this context, the amount of time during the winter below freezing likely influences *D*. *suzukii* overwintering mortality and the subsequent probability of early detection of flies in traps [65,66]. Summer temperatures and atmospheric moisture, however, have not been incorporated into a validated model previously, as it is unclear how prior summer conditions may affect the activity of overwintering *D*, *suzukii* adults and their survival into the next year though it has been found that higher capture rates have correlated within increased spring precipitation in other regions [64].

In contrast, since the invasion of *D*. *suzukii*, the seasonal populations of the native *R*. *mendax* have been declining in highbush blueberry farms in New Jersey. Like with *D*. *suzukii* data, this pattern was not explained by the climate variables we tested here. Also, *R*. *mendax* populations were not correlated with the amount of insecticide sprays or catch numbers of prior years. In a previous study [67], we showed that *R*. *mendax* trap captures in blueberry fields are positively correlated with higher insecticide sprays, indicating that blueberry growers target their sprays to fields that are at a higher risk of infestation. The discrepancies between the previous study and our current study could be due to the scale at which trap counts were analyzed; in our previous study, we analyzed trap data at the field level, while in this study, we used statewide data, which is likely less precise. Nevertheless, neither climate variables nor insecticide sprays seem to explain the decline in *R*. *mendax* populations since the invasion of *D*. *suzukii*. Instead, it is likely that the contrasting correlations between the *R*. *mendax* and *D*. *suzukii* seasonal catch numbers are due to *D*. *suzukii* outcompeting *R*. *mendax* for resources in the natural habitats surrounding blueberry farms–a potential case of competitive displacement [42,68]. Although *R mendax* has a narrower host range than *D*. *suzukii*, they both utilize wild blueberry (*Vaccinium* spp.) fruits in the understory of forests adjacent to blueberry farms [67,69]. The difference is that *D*. *suzukii* overwinters as adults and are active earlier in the year than *R*. *mendax* that overwinters as pupae with adults emerging later in the year. This difference in the biology and ecology of these pests gives *D*. *suzukii* a competitive advantage over *R*. *mendax*, possibly by enabling earlier access to host resources, which warrants further investigation. *Drosophila suzukii* also has a competitive advantage because it has multiple generations a year and lays multiple eggs per fruit [4], while *R*. *mendax* completes only one generation per year and lays a single egg per fruit [21]. Our assumption that *D*. *suzukii* is outcompeting *R*. *mendax* is anecdotally supported by the fact that *D*. *suzukii* has replaced *R*. *mendax* as the primary target and driver of insecticide sprays.

First *R*. *mendax* captures have been delayed since *D*. *suzukii* invasion. Following this cause-and-effect approach, the inverse correlation between the prior year’s first *D*. *suzukii* catch and the following years *R*. *mendax* first catch indicates that an increase in *D*. *suzukii* population numbers in one year (resulting from earlier activity) delays the first catch of *R*. *mendax* in the following year. In general, *R*. *mendax* first captures were less influenced by seasonal environmental conditions than *D*. *suzukii* first captures, which is understandable given that *D*. *suzukii* is an invasive species and might not be as adapted to the seasonal climatic stressors in New Jersey. The factors that correlate with the first catch days of *R*. *mendax* differed from those of *D*. *suzukii*, except for summer *T*_*dp*_. Notably, *R*. *mendax* first capture dates seem to be influenced by prior summer conditions, particularly those related to atmospheric moisture and rainfall. Prior to *D*. *suzukii* establishment, higher atmospheric moisture (indicated by *T*_*dp*_) in the prior summer was correlated with earlier *R*. *mendax* captures, while the trend reverses for summer rainfall after *D*. *suzukii* establishment. Since *R*. *mendax* pupates in the soil, soil moisture levels influence pupation depth [70], which can affect the exposure of pupae to natural enemies [71].

With climate change, the first capture date of *D*. *suzukii* is projected to be earlier in the year, coincident with warmer winter and summer temperatures and fewer degree days below freezing accumulated each year on average. The higher warming associated with RCP 8.5 (higher emissions) results in earlier capture by the middle of the century compared to the moderate warming of RCP 4.5. Therefore, it is reasonable to expect that while there may be significant year-to-year variation, *D*. *suzukii* will continue to be caught earlier on average, although the degree to which this effect is observed will depend on the level of warming, how the important environmental variables will evolve, and IPM practices moving forward.

The linear models themselves present differing trajectories for *D*. *suzukii* first catch dates, with the summer *DD*_50_ model presenting the greatest decrease in Julian day, the winter *DD*_32_ model presenting the smallest change, and the combined model falling between the two (Fig 5). This discrepancy can be linked to the differential warming in each season and how degree day values are calculated. Observed summer and winter temperatures have warmed at different rates within the region [72,73], with winters warming more rapidly than summers. This warming indicates that the magnitude of winter *DD*_32_ is likely to taper off rapidly into the midcentury (as there will be less time spent below freezing), somewhat stabilizing *D*. *suzukii* mortality from overwintering earlier in the timeline and resulting in a smaller change. The summer *DD*_50_ calculation does not include an upper threshold for which summer conditions may become detrimental for *D*. *suzukii* and is not a quantification of time spent in adverse temperatures for *D*. *suzukii* like the winter *DD*_32_ parameter describes. Temperatures above 30–32°C (86–89.6°F) have been found to be lethal for *D*. *suzukii* [74]. In using 31°C (87.8°F) as an upper limit in the degree day calculation, future projections did not differ substantially from an unconstrained degree day calculation (less than 1 day difference), and in practice, the projected average daily temperature rarely exceeded this threshold. The result is that the magnitude of the projected increase of summer *DD*_50_ is much larger than the decrease in winter *DD*_32_ by 2050, resulting in the single linear models providing differing first capture date projections. It is expected that the combined winter *DD*_32_ + summer *DD*_50_ model provides a better approximation of future capture dates by incorporating both effects, despite the wider confidence bounds. However, future improvements in these models should consider other environmental factors that may affect *D*. *suzukii* mortality, such as changes to humidity and heatwaves in the future.

There are additional caveats with our modeling approach. First, the winter *DD*_32,_ summer *DD*_50_, and combined *DD*_32_ + summer *DD*_50_ models are trained on a short dataset of 8 years. During this time, the conditions necessary to simulate potential future climate conditions may not arise (i.e., the future warmer winter and summer temperatures have not been observed), leading to model uncertainty. The limited number of training data points and their associated environmental conditions lead to larger confidence bounds by 2050 (Table 5), especially for RCP 8.5, which describes warmer conditions far outside the observed record. Additionally, in the observed data, the total range of first capture dates spanned 29 days, which is a wide range compared to the mostly modest projected changes. The few calibration data points with a wide range of first capture dates result in a less robust model, necessitating wide confidence bounds. Therefore, these projections should be used primarily as general guidance; on average, the state of New Jersey and others with similar climate conditions should expect to see *D*. *suzukii* appearing earlier through 2050 without pest management interventions, but that shift will be accompanied by wide year-to-year variation.

## Conclusions

Comparing the capture trends and population proxies for the invasive *D*. *suzukii* and the native *R*. *mendax*, we provide evidence that the introduction of *D*. *suzukii* and its subsequent establishment are the most likely causes for the decline in catch numbers and later first catch date of *R*. *mendax* each year. The first catch date of *R*. *mendax* is inversely correlated with the first catch date of *D*. *suzukii* of the prior year, with an earlier *D*. *suzukii* catch date delaying *R*. *mendax* first catch each year, resulting in greater populations and activity of *D*. *suzukii* adults through the seasons. According to these findings, we speculate that *D*. *suzukii* is outcompeting *R*. *mendax* for resources. Taken as a whole, the invasive *D*. *suzukii* has become more successful, while *R*. *mendax* has become less successful in recent years.

Seasonal meteorological variables more consistently correlated with *D*. *suzukii* population parameters as opposed to *R*. *mendax*. Temperature trends seem to favor *D*. *suzukii*, with higher summer temperatures and fewer winter days below freezing causing earlier *D*. *suzukii* adult activity and a larger population size. This is likely due to more intense winter conditions reducing the number of *D*. *suzukii* individuals successfully overwintering. It is expected that with climate change these trends will continue, i.e., the number of summer growing degree days is likely to increase and winter degree days below freezing are likely to decrease. Both of these conditions are correlated with greater *D*. *suzukii* activity and earlier first capture dates. Simple linear models project mostly modest earlier first capture dates of *D*. *suzukii* through 2050 on the scale of 1–2 weeks. While this change in the average capture date may be somewhat small, it strongly indicates earlier *D*. *suzukii* captures with climate change. Given that *D*. *suzukii* activity is the likely driver for *R*. *mendax* capture trends, with continued warming, *R*. *mendax* first capture will likely be later in the year and the capture numbers will continue to decline.

These findings have important implications for IPM in blueberries. At the beginning of the *D*. *suzukii* invasion, early-season blueberry varieties were relatively unaffected by this pest. However, as *D*. *suzukii* has appeared earlier with time, insecticide sprays increased in these early-season varieties. It is possible that classical biological control could change the trend predicted for *D*. *suzukii*. For instance, *Ganaspis brasiliensis* (von Ihering), a parasitoid of *D*. *suzukii* from Asia, has recently been approved for release in parts of North America and Europe [75]. This biological control agent is now being released in noncrop habitats surrounding commercial blueberry farms in New Jersey. These noncrop habitats serve as overwintering sites for *D*. *suzukii* and contain susceptible wild hosts [76]. If they are successfully established, we expect that *G*. *brasiliensis* or other introduced natural enemies may regulate *D*. *suzukii* in these noncrop habitats, changing the increasing trend in *D*. *suzukii* populations. Thus, biological control could be an IPM tactic that delays the appearance of *D*. *suzukii* in blueberry fields in colder regions in northern latitudes, such as New Jersey.
